# Seroprevalence of bluetongue in ruminants of Jharkhand

**DOI:** 10.14202/vetworld.2015.346-349

**Published:** 2015-03-18

**Authors:** Pinky Tigga, Siddhartha Narayan Joardar, Arkendu Halder, Chandan Lodh, Indranil Samanta, Devi Prasad Isore, Kunal Batabyal, Samir Dey

**Affiliations:** 1 Department of Veterinary Microbiology, West Bengal University of Animal and Fishery Sciences, Belgachia, Kolkata - 700 037, West Bengal, India; 2 Department of Veterinary Medicine, Ethics and Jurisprudence, Faculty of Veterinary and Animal Sciences, Belgachia, Kolkata - 700 037, West Bengal, India

**Keywords:** antibodies, bluetongue, indirect enzyme linked immunosorbent assay, Jharkhand, seroprevalence, virus

## Abstract

**Aim:**

This study was carried out to assess the presence of anti-bluetongue (BT) antibodies in sheep, goat and cattle of different agro-climatic zones of Jharkhand.

**Materials and Methods:**

Serum samples were collected from apparently healthy as well as suspected sheep, goat and cattle from different districts of Jharkhand covering different agro-climatic zones. Serum samples were screened by indirect enzyme linked immunosorbent assay (iELISA) for detecting anti-BT antibodies.

**Results:**

Out of a total of 480 animal serum samples (sheep-190, goats-210 and cattle-80) screened, 83 (43.68%) of sheep, 91 (43.33%) of goat and 46 (57.50%) of cattle sera were found positive. The % positivity ranged between 41% and 51% in different agro-climatic zones. The results showed slight higher seroprevalence, although not significantly, in cattle than sheep and goats in different agro-climatic zones of Jharkhand.

**Conclusions:**

The above data indicate widespread prevalence of BT virus antibodies in studied areas. The incidence of BT is not detected officially, so far. The present seroprevalence status of BT in Jharkhand indicates presence of BT infection in the state for the first time.

## Introduction

Bluetongue (BT) is an infectious, non-contagious, vector-borne viral disease that affects wild and domestic ruminants such as sheep, goats, cattle, buffaloes, deer and various other artiodactyla as vertebrate hosts. Cattle and goats are major vertebrate hosts of the virus, but sheep and deer usually exhibit clinical disease characterized by fever, depression, nasal discharge, drooling of saliva, oral lesion, facial edema, hyperemia of coronary bands and muscle weakness [1]. Bluetongue virus (BTV) is only enzootic in areas where continuous series of virus infection cycles in vector and vertebrate host are maintained. In ruminants, it may cause severe systemic disorders with moderate to high mortality. The infected bovines exhibit prolonged viremia compared with sheep and may act as reservoir the host for BTV [2].

While BTV is endemic in India, some strains inflict substantial numbers of clinical cases each year. Flocks are also at risk from regular hyper-endemic outbreaks, which the researchers believe could be caused by the annual monsoon. During one of these outbreaks, BT can kill 30% of sheep in a flock. Severe disease most commonly occurs in certain breeds of sheep, but the severity of BT is highly variable, ranging from sub-clinical to severe depending on virus strain and host susceptibility [3].

BTV is a member of the genus Orbivirus in the family Reoviridae. Its genome consists of ten double-stranded RNA segments coding for seven structural proteins (VP1-VP7) and four non-structural proteins (NS1-NS3 or NS3A and NS4). At present, 26 serotypes have been reported throughout the world [4]. Twenty-one out of 26 serotypes (except 19, 22, 24, 25 and 26) have been reported from different states of India [5-7].

BTV is transmitted by biting of blood-feeding insect vectors of the genus *Culicoides* spp. (Diptera: Ceratopogonidae) [8]. Culicoides (the insect host) transmit BTV among susceptible ruminants, having become infected by feeding on viremic animals (the vertebrate host) [9]. BTV, the causative agent of BT disease of ruminants, has now identified on all continents except Antarctica [10]. The first outbreak of BT in India was recorded in 1964 among sheep and goats in Maharashtra State [11].

However, eastern and north-eastern parts of the country did not experience any outbreak or reported active disease. Similarly, Jharkhand being one of the eastern states of this country, the incidence of BT is not detected officially, so far. However, that does not warrant declaring the state as BT free; as always there exists a complex interaction between BTV, *Culicoides* midges (vector), susceptible hosts and environmental factors that finally give rise to active disease. In this situation, it is quite pertinent to explore this interaction in a holistic manner to reveal the prevalence of sub-clinical BT and circulating BTV in the state, if any.

With this background, the present study was undertaken to assess anti-bluetongue antibodies in ruminants of different districts covering different agro-climatic zones in Jharkhand as the first step to reveal prevalence of BT in the state.

## Materials and Methods

### Ethical approval

As per Committee for the Purpose of Control and Supervision of Experiments on Animals (CPCSEA) guidelines, study involving clinical samples does not require approval of Institute Animal Ethics Committee.

### Sera

Totally 480 numbers of serum samples were collected randomly from apparently healthy sheep (190), goat (210) and cattle (80) of different age group from different districts of Jharkhand encompassing various agro-climatic zones, *viz*. Central and North Eastern Plateau Zone, Western plateau and South Eastern plateau. The animals were of marginal farmers and were not maintained in organized farms. The blood samples were collected into vacutainer tubes without ethylene diamine tetra acetic acid (EDTA). From blood samples, sera was separated and stored at −20°C till use.

### Indirect enzyme linked immunosorbent assay (iELISA)

The test was performed as per De *et al*. with relative sensitivity and specificity of 97% and 96.12%, respectively [12]. The average of the optical density (OD) values of negative control was calculated and compared with the test OD values. The OD values of tests which were higher than twice of the average OD value of the negative controls were considered as positive samples for anti-bluetongue antibodies.

### Statistical analysis

Chi-square test at two degree of freedom (5%) for detection of significant difference between positive sera samples with species of animals and agro-climatic zone was performed in SPSS version 21 (SPSS Inc., Chicago, USA).

## Results and Discussion

Serum samples of ruminants (Sheep [n=190], goat [n=210] and cattle [n=80]) were collected randomly from different districts of Jharkhand state, *viz*. Chatra, Hazaribag, Ranchi, Khunti, Chaibasa, Jamshedpur, Gumla and Lohardaga covering various agro-climatic zones to conduct seroprevalence study with an objective to assess the prevalence of BTV. After screening the samples by iELISA, 83 (43.68%) were found positive for sheep, 91 (43.33%) were positive for goat and 46 (57.50%) were found positive for cattle (Table-1). The above data indicate that, cattle have slight higher seroprevalence than sheep and goats in different agro-climatic zones of Jharkhand, although no significant difference was observed between the sera samples of different species at two degree of freedom (5%). Out of total 480 serum sample screened, 220 (45.83%) were found positive. The % positivity ranged between 43 and 57% among above animals. Detection of virus specific antibody in animals indicates an indirect evidence of virus in that area [13,14]. This implies that the cattle population acts as major carrier of virus and thus plays an important role in its dissemination. Cattle are considered to be the reservoir hosts of BTV because the viremia is prolonged and the majority of infections are sub-clinical [15].

**Table-1:** Prevalence of ant-bluetongue antibodies in serum samples of ruminants in Jharkhand as assessed by iELISA.

Species	Number of samples collected	Number of samples tested	Positive samples	Percent positivity
Sheep	190	190	83	43.68*
Goat	210	210	91	43.33*
Cattle	80	80	46	57.50*
Total	480	480	220	45.83

* No significant difference at two degree of freedom (5%), iELISA=Indirect enzyme linked immunosorbent assay

In the present study, overall 45.83% seroprevalence of BTV group specific antibodies were detected in sheep, goat and cattle, which is somewhat similar to an earlier report where 43.77% seroprevalence was detected in cattle, sheep, and goat from Assam [16]. The present values were also in accordance with the result of Sreenivasulu and Subba Rao [14] and De *et al*. [12] who reported overall 42.31% seroprevalence in Andhra Pradesh and 47% seroprevalence in sunderban area of West Bengal, respectively. However, still higher seroprevalence was reported by some earlier workers. Dayakar *et al*. [17] observed 71.43% seroprevalence in three states of South India, with 65.19% in Andhra Pradesh, 79.5% in Karnataka and 80.95% in Tamil Nadu using competitive ELISA (cELISA). Panda *et al*. [18] reported 60.26% seroprevalence using iELISA from West Bengal.

In case of sheep, the seroprevalence observed was 43.68% in the present study. The present values are in accordance with the result of Shlash *et al*. [19] who found 43.97% seropositivity using cELISA in Iraq. However, Naresh and Prasad [20] reported a much lower (23.5%) seroprevalence from Haryana, Himachal Pradesh and Punjab. The present data also differs from previous studies. Chauhan *et al*. [21] reported 36.11% seropositivity in sheep from Gujarat. Panda *et al*. [18] found 79 (57.66%) samples positive out of 137 sheep serum of West Bengal by iELISA. This shows that seroprevalence of BT in sheep varies from state to state.

In goat, about 43.33% seroprevalence was detected in the present study. This has got resemblance with the findings from Singh *et al*. [22] where 45.0% goats were found seropositive in Udhampur district, Jammu province. De *et al*. [12] described 47% seroprevalence in Goat while testing 1202 number of serum samples in Sunderban area of West Bengal using iELISA. When Panda *et al*. [18] conducted the seroprevalence study on BTV; the prevalence was found to be 66.95% in goat. However, Joardar *et al*. [16] reported 31.79% seroprevalence in goat of Assam after performing iELISA. This again shows wide variation in seroprevalence in goat of different Indian states.

In case of cattle, the seropositivity detected was 57.50%. This is similar to the observation of Panda *et al*. [18] who reported that cattle were 52.00% seropositive in West Bengal. However, Dayakar *et al*. [17] reported much higher seropositivity like 65.91% in Andhra Pradesh, 79.15% in Karnataka and 80.95% in Tamil Nadu. Joardar *et al*. [16] reported the prevalence of anti-bluetongue antibodies to be 70.00% in cattle serum samples of Assam while performing i-ELISA. However, considerable low seropositivity (2.69%) was reported in dairy Holstein cattle of Central Iran [23]. Variation of data regarding seroprevalence might be due to several physical factors (meteorological parameters), nature of virus (serotypes), as also breeds of animals.

In this present study, three agro-climatic zones were covered for sero-surveillance of anti-BT, *viz*. Central and North Eastern Plateau Zone, Western plateau and South Eastern Plateau. From three zones, a total of 347, 67 and 66 sera tested; 159(45.82%), 34(50.74%) and 27(40.90%) were found positive, respectively. No significant difference was observed between the sera samples of different agro-climatic zones at two degree of freedom (5%). The seroprevalence ranged between 41 and 51% in different zones, which indicates presence of circulating virus in all agro-climatic zones of Jharkhand (Table-2).

**Table-2:** Agro-climatic zone-wise seroprevalence of bluetongue in Jharkhand as assessed by iELISA.

Agro-climatic zone	Districts covered	Number tested	Number positive	Percent positivity
Central and North Eastern plateau zone	Chatra, Hazaribag, Ranchi and Khunti	347	159	45.82*
South Eastern plateau	Jamshedpur and Chaibasa	67	34	50.74*
Western plateau	Lohardaga and Gumla	66	27	40.90*
Total	480	480	220	45.83

* No significant difference at two degree of freedom (5%), iELISA=Indirect enzyme linked immunosorbent assay

## Conclusion

Incidence in sheep, goat and cattle being not reported so far, the present seroprevalence status of BT in Jharkhand is the first record of its kind. This study reflected high incidence of seroprevalence of BT infection in cattle, sheep and goats in this eastern Indian state. The results indicate that further studies are needed to identify the vector from different agro-climatic zones of Jharkhand and to determine the BTV serotypes that are circulating in Jharkhand.

## Author’s Contribution

PT, SNJ and AH implemented the study design and carried out the experiment. CL, IS, and DPI analyzed the data. PT, KB and SD drafted and revised the manuscript. All authors read and approved the manuscript.
